# Shiga Toxin 2A–Encoding Bacteriophages in Enteroaggregative *Escherichia coli* O104:H4 Strains

**DOI:** 10.3201/eid2009.131373

**Published:** 2014-09

**Authors:** Lothar Beutin, Jens A. Hammerl, Jochen Reetz, Eckhard Strauch

**Affiliations:** Federal Institute for Risk Assessment, Berlin, Germany

**Keywords:** Shiga toxin, bacteriophages, enteroaggregative, Escherichia coli, O104:H4, bacteria, E. coli, STEC, EAEC, outbreak, Europe, subunit A, Shiga toxin 2A, Stx-2, Stx-2A

**To the Editor:** In 2011, enteroaggregative *Escherichia coli* (EAEC) O104:H4 strains that produce Shiga toxins (EAEC-STEC) caused an outbreak of hemorrhagic disease affecting nearly 4,000 patients in Europe (*1*). During 2001–2013, several countries reported infections caused by EAEC O104:H4 and EAEC-STEC O104:H4 strains (*1*–*9*). Genomic analysis of EAEC and EAEC-STEC O104:H4 strains revealed high similarity, and it has been suggested that EAEC-STEC O104:H4 strains evolved from EAEC O104:H4 strains by uptake of Shiga toxin 2 (Stx2)–producing bacteriophages (*3*,*4*).

We investigated Stx-2 subunit A (Stx-2A) bacteriophages in a group of epidemiologically unrelated EAEC-STEC O104:H4 strains isolated from animals and food in Germany (collection of the National Reference Laboratory for *Escherichia coli*). One phage genome (P13374) was sequenced (*2*). The Stx-2A bacteriophages were highly similar in morphologic features, restriction endonuclease profiles, chromosomal integration sites, and superinfection immunity (*2*,*3*) and showed <65% similarity to Stx phages from non-O104 strains. Major genetic differences between the bacteriophages we investigated and other Stx phages were found in the genes for DNA replication, DNA metabolism, and in the immunity region (*2*,*3*).

We identified 2 genes, *orf15* and *cI*_P13374_, that were specific to Stx-2A bacteriophages found in EAEC-STEC O104:H4 strains (*10*). These genes were found in only 14 (5.8%) of 241 Stx-2A–positive non-O104 STEC strains. Viable Stx-2A bacteriophages isolated from 4 bovine non-O104 STEC strains were similar to Stx-2A bacteriophages from EAEC-STEC O104:H4 strains for all features described above (*10*). Similar to P13374, one of the bovine phages (P13803) lysogenized an Stx-negative EAEC O104:H4 strain and converted it into an EAEC-STEC–producing Stx-2A bacteriophage (*10*).

Our results provide experimental evidence that EAEC-STEC O104:H4 have evolved by uptake of a distinct type of Stx-2A bacteriophage. Bovine STEC harboring Stx-2A bacteriophages able to transduce Stx-2A genes to EAEC O104:H4 are found worldwide, and phage-mediated transfer of Stx-2A can occur in the environment (*10*). Thus, the emergence of EAEC-STEC O104:H4 does not appear to be the result of introduction of the strains from areas to which they are endemic. Instead, the process may have occurred spontaneously by phage transduction, which could explain why EAEC-STEC O104:H4 infections were found at different locations and at different times. Regardless of time or place, however, these strains show characteristic differences in their prophage and plasmid profiles, which may serve as indicators of epidemiologic origin (*1**–**4*). Investigation of EAEC-STEC O104:H4 strains from sporadic cases of human infection could reveal these markers and help differentiate between strains that were introduced from other areas and strains that were newly generated by phage transduction. 
